# Impact of the COVID-19 Pandemic on Mental Health and Lifestyle in Thai Occupational Therapy Students: A Mixed Method Study

**DOI:** 10.3390/ejihpe12110118

**Published:** 2022-11-18

**Authors:** Tiam Srikhamjak, Kanyarak Yanawuth, Kornkamon Sucharittham, Chitsanucha Larprabang, Patcharaporn Wangsattabongkot, Tanyathorn Hauwadhanasuk, Chirathip Thawisuk, Peeradech Thichanpiang, Anuchart Kaunnil

**Affiliations:** 1 Department of Occupational Therapy, Faculty of Associated Medical Sciences, Chiang Mai University, Chiang Mai 50200, Thailand; 2 Department of Sociology and Anthropology, College of Arts and Sciences, Saint Louis University, Saint Louis, MO 63108, USA; 3 Department of Occupational Therapy, Graduate School of Human Health Sciences, Tokyo Metropolitan University, Tokyo 116-8551, Japan; 4 Division of Occupational Therapy, Faculty of Physical Therapy, Mahidol University, Nakhon Pathom 73170, Thailand

**Keywords:** lifestyle, occupational therapy students, COVID-19

## Abstract

The impacts of the COVID-19 pandemic have led to global reports of hazards to mental health. However, reports regarding lifestyle changes due to the COVID-19 pandemic are lacking. Using a convergent mixed methods design, we conducted individual interviews with twelve occupational therapy students and interpreted the results by content analysis. We completed a survey of Thai Sensory Patterns Assessment (TSPA) concerning perspectives from occupational therapy students (*n* = 99). They identified two major themes: (i) adaptive responses were consistent with areas of occupation during the COVID-19 pandemic; (ii) multidimensional challenges were related to sensory patterns of purposeful and meaningful activities. The participants reported both positive and negative impacts of the COVID-19 pandemic on their lives. It had both positive and negative effects on the lifestyle of students affected by the COVID-19 pandemic. The positive effect was that most students learned better ways to protect and care for themselves. During the COVID-19 pandemic, occupational therapy students were most concerned about their online learning activities, economic problems, isolation from society, and lifestyle. The negative effects of this include stress, anxiety, loneliness, frustration, boredom, and exhaustion for occupational therapy students. As an impact of the COVID-19 pandemic, occupational therapy students adapted to new lifestyles and experienced mental health issues related to their studies, families, friends, economics, social climate, and future job opportunities. Educators may use the findings of this study to prevent negative impacts on mental health and promote academic achievement in the future, as well as general well-being, efficacy, and empowerment of students in the new normal post-COVID-19 pandemic era.

## 1. Introduction

The COVID-19 pandemic has resulted in the drastic loss of human life worldwide, and has presented unprecedented challenges for public health, food systems, and the world of work, including the education system [1]. COVID-19 has threatened student lifestyles. This pandemic has left people fearing for their safety and well-being, and campuses have been no exception. With classes cancelled, students struggling to access food and water, and dormitories overflowing, the student lifestyle has considerably changed. The spread of COVID-19 has had many impacts on people with mental health conditions and on mental health care globally [2]. Within this context, the main concerns of and changes faced by students are mental health, sleep, stress, learning environment, technology use, food, study habits, and social life. Students need to be supported in coping with the impacts of COVID-19; universities need to provide support and resources that are tailored to the unique needs of students. The stress and self-efficacy experienced by students should be considered by healthcare providers, families, and educators when devising interventions to encourage healthy physical and psychological behaviours in university students [3].

The new lifestyle and new normal due to COVID-19 have impacted on the university and student lifestyle in Asia [4]. The COVID-19 pandemic has led to several lifestyle changes, both within and outside the university environment. Amongst students, many have found that their social lives have shrunk, as forms of communication such as messaging and social media have become less effective. In addition, those who have been most affected by the virus are students, who are least likely to maintain healthy lifestyle habits, which leads to increased levels of anxiety and stress, which affects academic performance. Universities and colleges were already struggling to keep up with the ever-changing demands of the modern world; they are now also affected by changes due to the COVID-19 pandemic [5]. One such challenge is to balance the need for students to stay healthy and safe with their need for freedom and flexibility. Whereas some universities have implemented rigid policies that restrict students’ movements, others have tried to find a balance that allows for both safety and freedom [6].

Way of life involves people’s daily activities that have become routine or habit [7]. Neuroscientists think that lifestyle is caused by a nervous system mechanism that adjusts the circadian rhythms of the body, also known as the biological clock (such as the sleep–wake cycle, hormonal cycles, etc.), to environmental conditions (such as day, night, month, seasons, and year) [8]. Circadian rhythms and sleep disruption contribute to cell dysfunction if the body is unable to generate energy to repair and create new cells to replace dead cells [9]. This disruption leads to numerous chronic diseases [10]. To address these lifestyle issues, occupational therapy focuses on meaningful and purposeful human activities that are essential to health, comprising the core aim of the profession: helping people of all ages, including people with disabilities and limitations, perform everyday tasks or occupations [11,12].

Kielhofner [13] described the model of human occupation (MOHO) in terms of three levels of individual functional states in everyday life or lifestyle: occupational identity (recognizing who you are and what you want), occupational competence (participating in a range of occupations to meet the standard expectations of social norms and to sustain a satisfying pattern of occupational behaviour), and occupational adaptations (involving one’s adaptive response to meet an occupational challenge [14]. Kielhofner’s MOHO describes the three components of lifestyle—volition, habituation, performance capacity—which involve environment and lifestyle activities [13]. Volition refers to the motivation for occupation and the power to make one’s own decisions with interest, personal causation, and values. Habituation is the process of organizing occupations and routines. Performance capacity refers to the physical and mental abilities that underlie skilled engagement in occupations (such as musculoskeletal, neurological, and cardiopulmonary systems, and cognitive or mental processes, such as memory, planning, etc.) [13]. Kielhofner’s findings complemented those of Smith et al. [15] in terms of the aforementioned three components of lifestyles, which were found to be associated with life satisfaction. Time spent at work and leisure more positively correlated with the levels of life satisfaction than routines.

Velde and Fidler [16] designed the lifestyle performance model as a model of occupational therapy practices that emphasizes the power of occupation. They defined lifestyle as a component of a person’s total activity repertoire within the context relevant to the needs of the individual and the sociocultural norms of the society in which the person lives. They stated that each person has the ability to consistently develop their own lifestyle performance to attain a good-quality life through four domains of activities: self-care and self-maintenance, intrinsic gratification, social contribution, and reciprocal interpersonal relatedness. In the promotion of wellness, prevention and occupational therapy services are based on the four domains of activities of the lifestyle performance model, which are essentially centred around the individual’s daily lifestyle activities.

Presently, Thailand has only three occupational therapy school providers. The first occupational therapy educational program was founded in 1980 at Chiang Mai University in northern Thailand [17]; the second school was established in 2008 at Mahidol University in Bangkok, the capital city of Thailand [18]. Now, the third school has been established by the Srinakharinwirot University and the Department of Medical Services, Ministry of Public Health, which enrolled the first cohort of occupational therapy students in 2022 [19]. Occupational therapists are a profession that works with individuals who are having difficulty in performing daily activities and meaningful occupations. Occupational therapists help people with various conditions by conducting assessments and designing individualized treatments [12]. Occupational therapy students require a lot of knowledge about human movement and body functions, psychosocial functions, development from birth until adulthood, health as well as theories, frames of reference, models related to health and well-being. As the progress through this program, they gain invaluable clinical experience in an outpatient setting as well as internships at hospitals, rehabilitation centres and communities throughout Thailand. The outbreak of the COVID-19 pandemic has created many challenges for occupational therapy students as they learned their studies with working from home and caring for their families. This has led to increased levels of stress and anxiety among the students. Moreover, the outbreak of COVID-19 has led to a change in their lifestyle and mental health, which may have an impact on their career and future. Because of this, it is important for educational executives and occupational therapy educators to be aware of the potential impact of the pandemic on the mental health and lifestyle of occupational therapy students so that they can provide support and resources to help them cope with the challenges they may face.

However, relatively little research has focused on new lifestyles. Humans are occupational beings; thus, participating in everyday activities contributes to good health [20]. However, many occupational therapy students are struggling to find the right path after their graduation amid the COVID-19 pandemic. The outbreak of the COVID-19 pandemic has led to an unprecedented global health crisis, with significant implications for mental health. The mental health of occupational therapy students has been negatively affected by the outbreak of the pandemic. In addition to the classroom and clinic experiences, occupational therapy students in Thailand were also required to complete a clinical practice. The purpose of the clinical fieldwork is to integrate the students’ current knowledge with real-world experiences. It may lead to significant changes in the way of their thought related to clinical skills and work performance. Therefore, the authors conducted this pilot study with a study group of undergraduate students enrolled in an occupational therapy program at Chiang Mai University to explore the impact of COVID-19 in occupational therapy students.

### Purpose of the Study

There are very few studies regarding the new normal lifestyle of Thai students in occupational therapy programs during the COVID-19 pandemic. Occupational therapy students will become occupational therapists who play a vital role in promoting health and preventing disease and disability. Hence, it is important to know how occupational therapy students adapted themselves to a new lifestyle during the COVID-19 pandemic. The specific objectives of this study are:

⃋ To investigate the impact of the COVID-19 pandemic on the mental health and lifestyle of occupational therapy students’ experiences.
⃋ To explore the activities and lifestyle of occupational therapy students’ perspectives during the COVID-19 pandemic.

## 2. Materials and Methods

This study addressed the exploration of experience and perspective of occupational therapy students in terms of their mental health and new normal lifestyle activities during the impact of COVID-19. This research received ethical approval from the Associated Medical Sciences Research Ethical Committees, Chiang Mai University, code AMSEC-63EX-065.

This study used a mixed method design to collect both survey data and interview responses from occupational therapy students. Mixed methods research combines qualitative and quantitative approaches to help answer the research question [21]. Participants were recruited participants between November 2020 and April 2021 during the COVID-19 pandemic. Based on location and ethical approval, this study used both convenience and purposive sampling by selecting participants attending the university [22].

**Figure 1:**
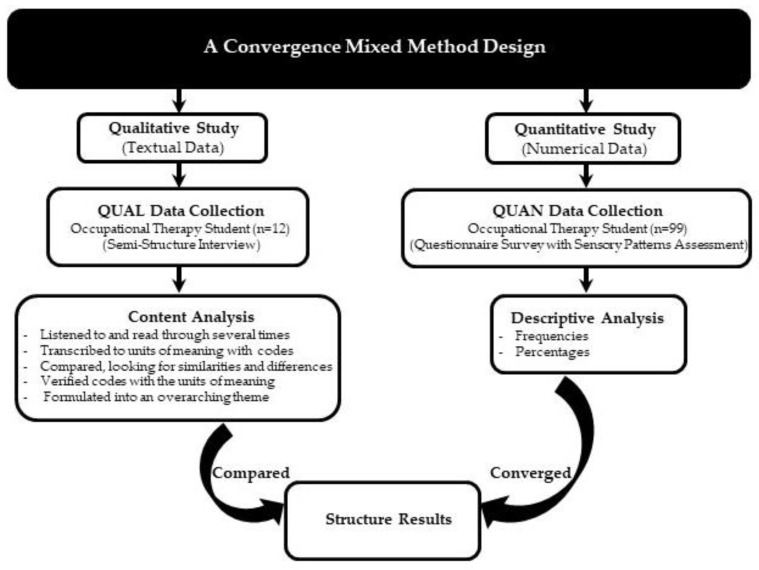
A convergent mixed method design [23].

A convergent mixed methods design was used [23], a type of design in which qualitative and quantitative data are collected in parallel, analysed separately, and then merged. In the qualitative inquiry, semi-structured qualitative interviews were conducted with 12 students (5 males and 7 females). In-depth interview data discovered the central phenomenon for occupational therapy students in their experiences in the response to individual adaptation, mental health, and new lifestyle activities. The interview data were analysed by content analysis [24]. In contrast of the quantitative study, questionnaire surveys were used to explore everyday activities of occupational therapy students (*n* = 99), visual activities and auditory activities and meaningful activities during the COVID-19 pandemic. The survey data were analysed using descriptive statistics and frequencies. Based on the research design, the structured results of this study integrated textual (qualitative) and numerical (quantitative) data (Figure 1) to present the study findings as a combination of information with relationship and coherence.

### 2.1. Interview Procedure

According to Barriball and While [25], as a method of exploring perception and opinions, a semi-structured interview is well suited to elicit information and clarification from respondents about complex issues. A question may be sensitive and enable a clarification and more information to be sought. This study was designed and refined the interview guidelines by an iterative process involving mock individual in-depth interviews prior to its use. The semi-structured interviews comprised the questions provided in Table 1.

As the wordings and sequence of all the questions in a standardized interview schedule were the same for each respondent, the authors ensured that any differences in the answers were due to differences between the respondents rather than in the questions asked [26]. The interview protocol was validated with an expert review and pilot test to ensure the focus of the interview [27]. The data collection process was continued until data saturation, when it had enough information to replicate the study [28], when authors were no longer able to obtain additional new information, and when further coding was no longer feasible [29].

### 2.2. Interview Participants and Recruitment

Participants were recruited based on the following criteria: (1) occupational therapy student; (2) willing to participate in an interview. The individual participants were excluded from the survey to avoid data duplication. The recruitment advertising was presented to student accommodations and the learning buildings over a one-month period. After participants were contacted by the research team, they were selected based on the inclusion criteria.

### 2.3. Survey Procedure

A variety of sensory processing pattern assessments are currently used to implement healthcare services. The Thai Sensory Patterns Assessment (TSPA) tool was developed in Thailand by Tiam Srikhamjak and colleagues [30,31]. Dunn’s sensory profile can be adapted to Thai’s sensory patterns assessment (TSPA) tool for adolescents and adults in two parts: sensory preferences and sensory arousal. A sensory preference is defined as the behaviour you prefer to express in relation to a particular sensory stimulus in your daily life. An instance of sensory arousal occurs when a person responds impulsively to a particular sensory stimulus. In this previous study, a multistage sampling method was used to select the sample group. A total of 400 participants aged over 15 years in Chiang Mai province were recruited for examining internal consistency, and 40 participants were recruited for testing test–retest reliability. Using the TSPA tool is easy, and it can be used to link behaviour responses to sensory stimuli in a variety of ways. The previous study showed that the TSPA tool for adolescents and adults provided a feasible tool for identifying sensory preferences to match health promotion modalities appropriately [32].

The authors developed a questionnaire survey using TSPA for adolescents and adults, which consists of two modules: one for sensory preferences with 35 items and the second for sensory arousals with 25 items. Each module is divided into six categories according to the type of sensory modalities: sight, sound, smell and taste, touch, proprioceptive, and vestibular systems [32]. The content validity examination of the index of item–objective congruence (IOC) of the modules I and II ranged from 0.60 to 1.00. Authors assessed the internal consistency reliability of the questions, receiving an α coefficient of 0.89 for module I and 0.62 for module II. The test–retest reliability with intraclass correlation coefficient (ICC) method was 0.91 in module I and 0.92 in module II. In conclusion, it was found that the TSPA was both valid and reliable at an acceptable level.

The questionnaire survey included two parts of questions: (I) personal information; (II) taking the self-report assessment. The participants were instructed to read each question and then select their answer by marking a cross (X) in the box corresponding to the frequency of the six sensory neurological response behaviours that are normal in daily life with the following criteria: Never: never feel like or behave like this, where the behaviour is shown only 5% of the time; Seldom: rarely respond to the stimuli, where the behaviour is shown only 25% of the time; Occasionally: this behaviour often happens at least once per week, where the behaviour is shown 50% of the time; Frequently: this behaviour happens at least once per day, where the behaviour is shown 75% of the time; Always: feel like this behaviour happens at least several times per day. This sensory pattern reflected visual, auditory, smell/taste, and movement activities that are involved in activities of daily living during the COVID-19 pandemic. The domains and questions were used to explore the sensory patterns of occupational therapy students, as shown in Table 2.

### 2.4. Survey Participants and Recruitment

The inclusion criteria for the participants were undergraduate students who were enrolled in the occupational therapy program for the second semester of the academic year 2020, which included first-, second-, third-, and fourth-year students. The recruitment for study participation was sent through a student leader of each class year. Authors used sampling to identify and select information-rich cases related to the subject of interest in this study.

### 2.5. Data Collection

For the qualitative inquiry, the data were collected in December 2020–January 2021. Authors contacted the occupational therapy students based on the inclusion criteria to arrange the most convenient time and used Zoom to conduct the individual interviews. The interviews were utilized by a semi-structured interview guideline. Each participant was required to read and sign a written consent form before the interview. The interviews were audio-recorded and then transcribed verbatim. The in-depth interviews lasted approximately 45–60 min. Authors clearly documented all research processes, and the team peer reviewed the transcripts. This study used pseudonyms to protect the identity of the participants.

For the quantitative approach, there were 99 occupational therapy students who were willing to answer the questionnaire with the sensory pattern assessment form, demographic questionnaires, consent forms, cover letters, and stamped return envelopes in November and December 2020. All participants read and signed written consent forms before answering the questions.

### 2.6. Data Analysis

For the qualitative analysis, seven female and five male occupational therapy students (mean age = 20 years) participated in the individual interview. The participants were between 18 and 23 years of age across different years of study, as first- to fourth-year occupational therapy students, and all signed consent forms before entering the study. Pseudonyms were used to protect the identity of the participants. Characteristics of the participants are presented in Table 3.

Individual interview responses were analysed by content analysis [24]. This analytical process utilized five steps: (1) the interviews were listened to and thereafter read through several times to obtain a sense of the whole; (2) the transcribed text was divided into units of meaning, which were condensed and labelled with codes; (3) the codes were compared, looking for similarities and differences, and then sorted into subcategories and two main categories; (4) verified that the coding was congruent with the units of meaning; (5) the latent content was formulated into an overarching theme. Moreover, the interviews were analysed and interpreted data related to the living environment and the lifestyle in accordance with Kielhofner’s human occupation model [13] and the Velde and Fidler’s lifestyle performance model [16]. The triangulation methods validated the data by member checking. Authors organized, coded, and categorized the data by themes. Detailed summaries of each theme are described in the Results section.

For the quantitative analysis, descriptive statistics were used to analyse the data that were returned by the end of December 2020, which included frequencies and percentages. A questionnaire survey was also completed concerning occupational therapy students’ perspectives; these participants provided consent to participate in this study. The demographic information of the participants included students in the age range between 18 and 25 years of age (mean age = 20.75). The respondents were in an occupational therapy program (*n* = 99) that included males (26.3%, *n* = 26), and females (73.7%, *n* = 73). This was presented by first-year (30.3%, *n* = 30), second-year (23.2 %, *n* = 23), third-year (25.3%, *n* = 25), and fourth-year (21.2%, *n* = 21) occupational therapy students (Table 4.).

### 2.7. Rigor and Trustworthiness

The potential translation-related problems were examined that might have interfered with the trustworthiness of the results. The transcripts were analysed word-for-word in the native language (Thai) and then were translated into English [33]. These transcripts were checked by two academics fluent in Thai and English and then back-translated from English into Thai. While translating and analysing the data, they considered the challenges of translating. To maintain semantic equivalence with realistic and textual meanings, authors considered linguistic differences. For example, an expression in one language may not be present in another [34]. The trustworthiness created an audit trail to provide support for interpretations and analyses [35]. Adequate translation contributes to a study’s trustworthiness because it ensures accurate data, inclusivity, consistency, and transparency during the analytical process [36]. To increase data transferability, authors documented the process and peer-reviewed the transcripts. Three independent reviewers spent a large amount of time on data translation, demonstrating our rigor in this study. Moreover, we invited participants to validate the findings by confirming or suggesting variant meanings that could be incorporated into the final version.

## 3. Results

Two themes arose from the analysis of interview data: (1) adaptive responses and (2) multidimensional challenges (Economic impact; Online activities; Impact on lifestyle and social life impact). These themes were integrated with survey findings. Blended results provided themes which were supported by descriptive interpretations. Textual and numerical data provided outcomes related to the impact of the COVID-19 pandemic on mental health and lifestyle in Thai occupational therapy students (Table 5).

### 3.1. Adaptive Responses Were Consistent with Areas of Occupation during the COVID-19 Pandemic

#### 3.1.1. Adaptive Responses

This has been a time of great change. As the COVID-19 virus infection became more prevalent on campus, more people became aware of its risks and how to protect themselves. This opportunity was used to empower students with the knowledge on how to stay safe, which is evident in the following interview extracts:

“Now, I have become more health conscious and more careful. Wearing a mask and washing hands became my habits. After going out and returning home, I took a shower immediately.”

(Student 1)

“I feel like a mask has become a part of my body. I must wear it all the time. When I am eating, I must wash my hands first. When I was not feeling well, I paid more attention on my symptoms and visited the doctor with no hesitation. While in the past, I never cared about it.”

(Student 3)

“In the past, I didn’t really care about the sanitizing that much. When some foods dropped on the floor, I just picked it up and ate it. With the COVID-19 pandemic, I have to keep myself clean, wash my hands, and wear a mask because I am afraid of being infected.”

(Student 7)

“Now, I have to wear a mask and wash my hands often. So, I don’t get infected with COVID disease. I didn’t care about this before. Wherever I went, I didn’t care that much about sanitizing. It’s very different now.”

(Student 4)

Some have adjusted their self-care and financial management routines, including food, rest, exercise, and leisure activities by focusing on performing in-home activities instead of participating in social activities in public, as described below:

“I used to go out jogging every evening. But now, I must switch to indoor exercise; for examples, sit ups and lifting dumbbells because I’m afraid of being infected with the virus.”

(Student 5)

“Previously, I was always hurried to go to school and skipped the breakfast. So, I got gastritis. With the COVID-19 situation, I switched to take online classes. As a result, I could have breakfasted every day.”

(Student 12)

“I usually like to go out for adventure activities. When I got infected with COVID-19, I stayed at home and read comic books. I learned and searched for tourist information from traveling guidebooks. When the COVID-19 pandemic settles, I’ll be able to take a trip with my friends.”

(Student 8)

“I want to earn money and help remove part of the burden on my family. During the COVID-19 pandemic, I sold the clothes that I didn’t wear anymore on Facebook. It’s selling very well. A lot of people have bought my clothes.”

(Student 9)

“I stopped going out drinking with my friends. Because of studying at home, I saved some money for traveling and eating out.”

(Student 10)

These perspectives of occupational therapy students were supported with numerical data from the survey. The purpose was to ascertain the perspectives of participants in self-adaptation during COVID-19.

#### 3.1.2. Areas of Occupation and Daily Activities during the COVID-19 Pandemic

During the study period, all participants responded to these two questions: What do you do each day from wake-up time to bedtime? Is there a difference between what you normally do Monday through Friday, Saturday and Sunday? (If so, what?) Most students spent time focused on activity of daily living (ADL), leisure activities, and health management.

#### 3.1.3. Monday through Friday

**Figure 2:**
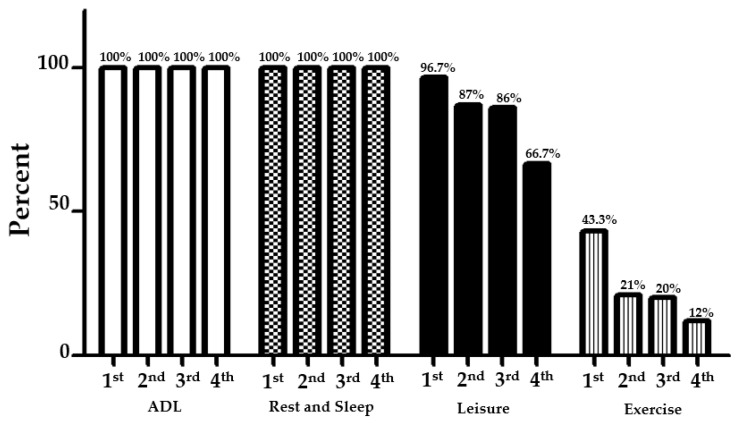
Activities of daily living during the COVID-19 pandemic: Monday through Friday.

Monday through Friday, participants (first- to fourth-year occupational therapy students) woke up to perform activities of daily living (ADL) and personal hygiene (100%), such as taking a shower, eating breakfast, ordering food through an application, having a lunch break, and having dinner. In rest and sleep, all participants had time to sleep (100%). In terms of leisure, participants spent time on phones and watched YouTube, movies, or TV series, read comic books or novels, and chatted with friends as different ways of spending their time (Figure 2).

Most of the first-year participants (96.7%) spent time on leisure activities. Second- and third-year occupational therapy students (87% and 86%, respectively) spent time on leisure activities, while only 66.7% of the fourth-year occupational therapy students did. Participants concerned themselves with exercise to maintain their health during the COVID-19 pandemic. Most participants in first-year occupational therapy (43.3%) spent their time concerning themselves about exercise. Moreover, second- and third-year occupational therapy students (21% and 20%, respectively) focused on their exercise. Only 12% of fourth-year occupational therapy students were concerned about spending time on exercise.

#### 3.1.4. Saturday and Sunday

**Figure 3:**
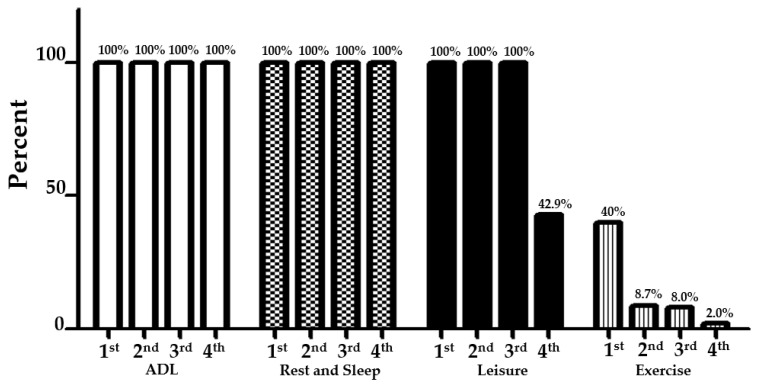
Activities of daily living during the COVID-19 pandemic: Saturday and Sunday.

Saturday and Sunday, participants (first- to fourth-year occupational therapy students) woke up to perform activities of daily living (ADL) (100%). After waking up, students reported that they took a shower and immediately dressed, had breakfast, lunch, and dinner (ordering food through applications). In rest and sleep, all participants had time to sleep (100%), with most of them going to bed around midnight on the weekend (Figure 3).

All first-, second- and third-year participants reported engaging in leisure activities on the weekend. They reported that they spent most of their time on their favourite social activity, alternating it with educational activities to avoid fatigue. In the evening, the students met with friends between their favourite activities such as reading novels, playing video games, and watching YouTube, movies, cartoons, and TV series. Only 42.9% of the fourth-year occupational therapy students participated in leisure activities on the weekend. The first-year participants were more than four times (40%) as likely to exercise on the weekends as the second- (8.7%) and third- (8%) year participants. Only 2% of fourth-year students reported exercising on the weekends.

### 3.2. Multidimensional Challenges Were Related to Sensory Patterns of Purposeful and Meaningful Activities

The COVID-19 pandemic had some negative impacts on the economy, educational models, and personal and social life, which gradually resulted in mental health problems.

#### 3.2.1. Economic Impact

The families of students were economically impacted as a result of the COVID-19 pandemic. Some students received less money from home and work, which caused stress. They provided the following responses:

“When COVID-19 occurred, my family business went bankrupt. My expenses have increased while my income has decreased. I am obviously under a lot of stress.”

(Student 4)

“I previously worked as a freelancer for a movie filming company. During COVID-19, there were no freelancing jobs at all. There were only jobs in the coffee shops, where I am now working for a lower wage with a person whom I know. My income has dropped a lot.”

(Student 5)

“Now, my savings earned previously are all gone. I have a tight budget. My mother has to also financially support my elder sister’s education.”

(Student 1)

#### 3.2.2. Online Learning Activities and Favourite Hobbies Were Related to Visual Activities

##### Online Learning Activities and Favourite Hobbies

In terms of online activities, most students reported that they spent time on the screen to study educational programs every day. In terms of favourite hobbies, they spent more time watching YouTube, movies, and TV programs and series, and playing computer games. Many activities had been changed and adapted such as traveling/photography, reviewing lesson content, drawing or colouring pictures and reading novels for a week, from morning to evening Monday through Friday, including the weekend. Participants reflected on the outcomes of online learning, as shown in the following comments:

“I have no concentration for studying and don’t understand the subject content. I would rather study in a classroom with the instructor standing in front of me so that I don’t feel sleepy.”

(Student 1)

“I don’t like studying online. My consciousness of learning has decreased with online learning. I don’t get what I learned. It made me feel sleepy. I prefer to study in the classroom.”

(Student 3)

“I didn’t learn anything from online classes. I can’t concentrate. The bed sucks me up and puts me to sleep. I think that it is better to study in the classroom than online because I can meet with friends. The instructors motivated me to learn when I felt sleepy.”

(Student 9)

“At first, I thought it would be fine to study online. But after studying for a while, I felt lazy, a lack of concentration, don’t understand what I have learned, and don’t get it into my head.”

(Student 10)

##### Visual Activities

**Figure 4:**
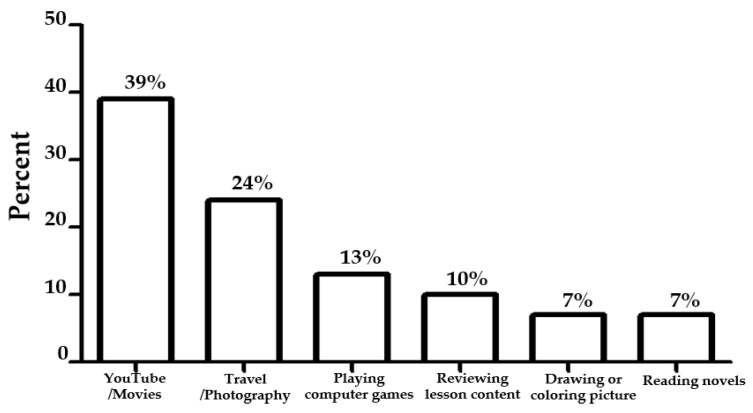
Visual activities enjoyed by participants.

As revealed in the above quotes, the impact of online learning showed that participants who were not familiar with this educational system were affected the most. However, the online and off-line activities from the survey had reflected the numerical data about spending time on visual activities (Figure 4).

Most participants reported that they enjoyed activities involving visual tasks. The result showed that 59 out of 99 participants enjoyed performing visual activities, such as watching YouTube/movies (39%), travel/photography (24%), playing computer games (13%), reviewing lesson content (10%), drawing or colouring pictures, and reading novels (7%).

#### 3.2.3. Impact on Lifestyle Was Related to Auditory, Smell/Taste, and Movement Activities

##### Impact on Lifestyle

In our daily lives, most of us tend to have certain habits as determining factors, such as eating, sleeping, studying, working, and social activities. Habits allow a person to be more agile and conserve energy when performing daily life activities. However, the COVID-19 pandemic has profoundly and globally affected all aspects of economic and social life. Governments have closed borders, banned mass gathering, and enforced social distancing, which have generated a new normal for business and individual citizens [37]. These measures have been implemented to protect public health but have threatened the global economy [37] and have changed people’s new lifestyles, and particularly the life of students in occupational therapy programs, which is highlighted in the following interview extracts:

“With COVID-19, my life has become more difficult. Before, I was able to sit and eat Shabu [hotpot] with my friends comfortably. But now I have to sit apart, wash my hands, and wear a mask often. It’s difficult.”

(Student 2)

“Wherever I go, I have to think more if the place I want to go is crowded with people. If that place is congested with people, I will not go. As a result, it appears that I was un-willing and worried to go anywhere. It was too much trouble in going anywhere. When I got back home, I needed to hurriedly take off my clothes, soak them in antiseptic deter-gent, and go take a shower.”

(Student 8)

“With COVID-19, I don’t dare to go anywhere and was afraid to go anywhere. I have to wear a mask all the time when going out. The current situation is totally different that I do not need to wear a mask and wash my hands so frequently.”

(Student 9)

“I have become an anxious and paranoid person from being forced to wear a mask, carrying and using alcohol hand sanitizer gel at all times, and having soreness caused by prolonged exposure to hand washing. Even if I order food, I need to spray alcohol all over the bag before carrying it.”

(Student 10)

##### Auditory, Smell/Taste, and Movement Activities

**Figure 5:**
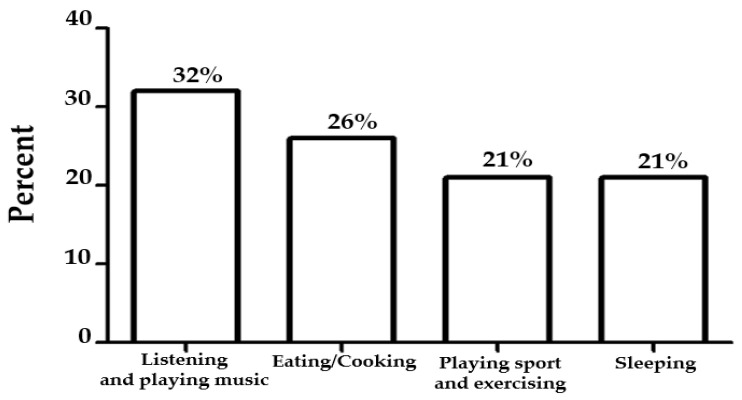
Auditory, smell/taste, and movement activities enjoyed by participants.

As revealed in the impact on lifestyle, these accounts showed that participants tried to protect themselves from COVID-19. They washed hands and wore masks in public areas for having meals and carried out activities with physical distancing. The impacts on lifestyle were integrated with auditory, smell/taste, and movement activities (Figure 5), of which participants staying at home or carrying out activities related to these sensory patterns.

In total, 36 out of 99 participants reported they enjoyed auditory activities (32%) by listening to and playing music and smell/taste activities (26%) by eating and cooking. Some participants preferred to remain active by playing sports and exercising (21%). In contrast, others spent time sleeping (21%).

#### 3.2.4. Impact on Social Life Was Appropriate for Meaningful Activities

##### Impact on Social Life

The continued spread of COVID-19 is having an increasingly negative impact on social lives. Many people are isolating themselves to avoid contact with others, which is leading to a decrease in social interactions and a rise in loneliness and isolation. In addition, restaurants and coffee shops have been closed. Many events and gatherings have been cancelled, which has further decreased opportunities for socializing. The pandemic is also causing stress and anxiety for many people, which can impact social life.

“It makes me feel lonely, uncomfortable, irritable, bored, and exhausted. It’s better to study in the classroom than online because I had my friends sitting next to me when studying in the classroom. The instructors engaged me in learning and made me to stay alert. When I had a question, I could ask my friend or the instructor. When studying online, I am on my own and am not eager to ask the instructor questions.”

(Student 1)

“I miss my life before COVID-19 a lot. In the past, I and my friends went to study at the coffee shop until 1:00 A.M. Now, the shop is closed at 11:00 P.M. I invited my friends to come along, but they are afraid of COVID-19 and refused to go study at the coffee shop with me like before. So, I go alone and feel lonely.”

(Student 9)

“As my friend and I planned to visit places when we have days off, we bought a lot of clothes and started matching the colour tones of our clothes in different sets for photos taken. We searched for places and nice coffee shops. Once COVID-19 emerged, our plans were cancelled. I could not meet my friends and travel. I miss my friends and traveling so much.”

(Student 11)

“I feel uncomfortable with the COVID-19 situation. I rarely see my close friends because we study in different programs and have time conflicts. I reached out to my academic advisor who suggested that I find any favourite activities to make me happy without going out. I tried to do various activities but could do them only for a short period of time and got bored. I ended up feeling stressed.”

(Student 4)

“All of my friends went home, and I had to stay alone in the dorm. I do not meet anyone except the people at the grocery stores. I wish the situation could have been the same as before because I would rather socialize with people.”

(Student 6)

##### Meaningful Activities

**Figure 6:**
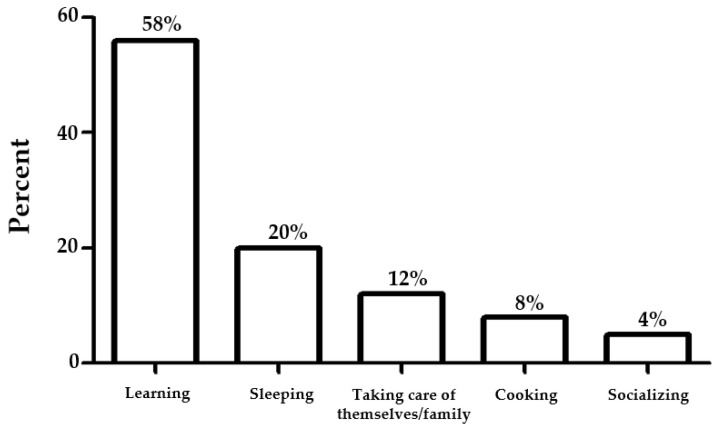
Meaningful activities enjoyed by participants.

These experiences of impact on lifestyle and social life were supported by numerical data from the survey (Figure 6). The purpose was to ascertain the perspectives of occupational therapy students during the COVID-19 impact on mental health and their lifestyle.

Most participants responded to survey about patterns of spending time on their activities. Over half of the participants valued learning activities (58%), sleeping (20%), taking care of themselves/family (12%), cooking/dining (8%), and socializing (4%).

## 4. Discussion

In this study, the time-based daily life patterns of the activities of the students were consistent with Kielhofner’s model of human occupation [13], in which students are motivated and patterned, and performed meaningful activities, particularly educational activities. Considering the lifestyle performance model of Velde and Fidler [16], the patterns of students’ daily life activities did not indicate a good quality of life as determined by the four domains of activities: self-care and self-maintenance, intrinsic gratification, social contribution, and reciprocal interpersonal relatedness. For example, most students woke up late on Monday through Friday, rushed to attend online classes, and did not take a shower or eat breakfast. During lunch time, they ordered food delivery using applications. Despite having spare time on Saturday and Sunday, students went to bed late and woke up late in the afternoon. They did not eat breakfast or perform activities for pleasure.

Students spent time on leisure activities Monday through Friday. They alternated spending time on leisure activities with educational activities on the weekend, afternoon and throughout the evening. Before bedtime, students likely spent time on passive activities (such as watching YouTube, movies, cartoons, or TV series; playing games on the computer; etc.) that required the use of visual sense, which they had been using throughout the week, instead of performing physical activities (such as sports and exercise). The responses showed that students (43.3% Monday–Friday, 40% Saturday–Sunday) in first year performed physical activities such as exercise. The rest of the students in other years of the program spent less time exercising because of the increased academic workload in the occupational therapy program.

First-year students spent more time participating in leisure and exercise activities than students in the other years of study. It was possible that this happened with first-year occupational therapy students in Thailand due to the COVID-19 pandemic. As the students graduated from high school and became university students, the Thai government promoted physical distancing and university-regulated online learning for them. As a result, they stayed home and could perform activities that they wanted to. Fourth-year occupational therapy students spent less time on leisure and exercise. The possibility of this occurring with this group was due to the fact that they were final-year occupational therapy students who were concerned about practical work and had to do a lot of homework. They may have also been worried about their future jobs. According to a study in England, nursing students who wish to undertake extended placements can do so from their homes if they cannot or do not wish to stay in university accommodations. In this period of rapid change, health services, government agencies, and educational providers will each have a different view of what these options might offer students in terms of benefits and risks [38]. Furthermore, nursing students in their final year were employed to provide nursing care during COVID-19. There were many concerns unresolved in their immediate futures. COVID-19 led to financial problems and job insecurity among university students in France [39]. It was found that the students most at risk were those who believed that the lockdown would negatively affect their employment prospects in the future [40].

In terms of students’ patterns of spending time on activities, we have concerns about some neuroscientific factors. The human body releases the hormones necessary for self-repair in response to changes in environmental conditions between day and night and having a lifestyle that is inconsistent with nature [41,42]. Most students had late sleep–wake patterns, which posed a higher risk of biological clock deviation, which can impact overall health status. Therefore, educational institutions should consider implementing proactive strategies to ensure preservation of mental health. For example, students on some online courses may feel isolated. The use of online working groups may benefit from initiatives to increase social interaction and share ideas, including the positive feedback by educators.

During COVID-19, students reported that they spent time on social media and chatting with friends. They had no concentration for online learning and did not understand the subject content. These negative impacts indicate the existence of a neuro-scientific phenomenon called habituation. Habituation is caused by a cluster of neurons that form the reticular activating system, which works in conjunction with the limbic and hypothalamus systems to modify the structure and function of the brain’s flexibility (neuroplasticity) by decreasing the response to repetitive stimuli as well as the connection between neurons or synapses [43,44]. As a result, alertness and focus on learning are reduced.

Two approaches can be applied to increase the response to stimuli, called sensitization, which can increase alertness and focus on learning [45]. The first approach is by providing the subjects a seemingly harmful stimulus or threats that lead to the release of stress-causing chemicals, such as teacher reprimands or various forms of punishment. This approach results in the short-term restructuring and functioning of the habit. The second approach is based on the flexibility of the brain as well but is more sustainable: experience-dependent plasticity. Successful experience leads to the release of rewarding chemicals, causing enthusiasm to repeat the same activity over and over until it becomes a habit [43]. Determining which approach to choose is an important question for educators when designing a pedagogy that promotes student engagement in online learning as well as increases student alertness and focus on learning.

In terms of the impact on social life, a critical review of Aristotle’s view on sociality [46] stated that humans are social animals: they flourish when in the company of others (“humans are social animals”, n.d.). They cannot exist without others. Psychologists and sociologists agree that one’s social life is one of the most important influences on mental and physical health. As a metaphor, human society is characterized as a spider web in which individuals interact with each other over the course of their lives. Such networks are an important source of training for various habits and skills. They are also sources of friends who have similar ideas and ideals with whom to converse, and exchange of information produces a sense of security, love, and being loved. However, measures for social distancing that keep people away from each other to prevent the spread of coronavirus cut off social networking and decrease social space.

Moreover, surveys were conducted before and during the COVID-19 pandemic in Iceland to determine the level of depression, mental well-being, and substance use among adolescents. Symptom Checklist-90, Short Warwick Edinburgh Mental Well-being Scale, and frequency of cigarette smoking, e-cigarette use, and alcohol consumption were used in this study. As a result of COVID-19, adolescent mental health is significantly impaired. However, it is possible that the decline in substance use observed during the pandemic might be an unintended benefit of isolation. This might serve as a protective factor against future substance abuse disorders. A population-level prevention effort is necessary, especially for girls [47]. According to the study in Turkey, there was a study about the social support and psychological well-being of adolescents during the COVID-19 pandemic. There was a positive correlation between the scores for social support and psychological well-being. The study found that the levels of social support perceived by the adolescents were decent, even during the outbreak of COVID-19. The study showed that social support among adolescents increased, and their psychological well-being could be improved positively [48].

Consequently, the data of this study were consistent with the results reported by Xiong et al. [2] regarding the impacts of COVID-19 on the sample population groups in eight countries, including China, Spain, Iran, the United States of America, Turkey, Nepal, and Denmark. The authors concluded that the COVID-19 pandemic had posed an unprecedented threat to students’ mental health, particularly with anxiety, depression, as well as emotional and general stress. The most high-risk population was found to be female with student status, younger than 40 years of age, with chronic mental illness and exposure to social media and COVID-19 news.

## 5. Limitations

In this study, the authors found that occupational therapy students changed their lifestyle during COVID-19, which was related to their mindset and attitude toward adaptation. First, the nature of human life is diverse, complex, and dynamic. To understand the daily lives of the study participants, the authors collected a wide variety of information from various data sources. Due to the time constraints and conditions, people had to stay apart. The authors therefore collected data through semi-structured interviews via different online social media channels, which should be studied further in the future. Social media platforms are subjective channels, and this may distort information accuracy. Secondly, the study participants were limited to occupational therapy students at universities in Thailand. Lastly, the authors studied only one university’s occupational therapy program; future studies should investigate other Thai universities that produce occupational therapists.

## 6. Conclusions

In summary, the COVID-19 pandemic has had both positive and negative effects on occupational therapy students’ lifestyles. Most students learned how to better protect and take care of themselves. In adaptive responses, students adapted themselves to engage in daily activities. They usually performed activities of daily living, rest, and sleep on a daily basis. However, they spent less time on exercise, and leisure activities were various depending on the year of the students. In multidimensional challenges, students faced economic impact, online activities, and impact on lifestyle and social life. It negatively affected students and, consequently, their emotional state and mental health problems. The COVID-19 pandemic led to a new normal for students involving more online learning activities, isolation from society, and financial crises. Their lifestyle choices also changed as a result of the pandemic. Students have been forced to confront many challenges that have taken a toll on their mental health. This has led to the negative effect that students faced with stress, anxiety, loneliness, frustration, boredom, and exhaustion. To support students with mental health challenges, university executives and educators should integrate agencies to strengthen students’ relationships and promote social–emotional learning and adaptation. They may use the findings of this study to prevent negative impacts on mental health and encourage academic achievement in the future, as well as general well-being, efficacy, and empowerment of students in the new normal post-COVID-19 pandemic era.

## Figures and Tables

**Table 1:** Interview questions.

Interview Guideline
(i) What do you usually enjoy doing the most? What is the most important to you? What do you do the best? (ii) What is your routine schedule from waking up to bedtime? (iii) Is there a difference between what you normally do between Monday through Friday, Saturday and Sunday? If so, what is the difference? (iv) After the start of the COVID-19 pandemic, how has your life changed?

**Table 2:** Research questions in the survey.

Domains to Pattern	Questions
Domain I: Sensory patterns of feelings and thoughts	(i) What do you enjoy doing the most?
	(ii) What activity is the most important to you?
	(iii) What activity are you doing the best?
Domain II: Lifestyle patterns of students in doing	(iv) Monday through Friday
activities during the COVID-19 pandemic	(v) Saturday and Sunday

**Table 3:** Demographic characteristics of participants in the interview (*n* = 12).

Participant	Gender	Age	Year of Study
Student 1	Female	19	2nd Year
Student 2	Male	18	1st Year
Student 3	Female	19	2nd Year
Student 4	Male	20	3rd Year
Student 5	Male	19	2nd Year
Student 6	Male	21	4th Year
Student 7	Female	19	2nd Year
Student 8	Female	23	4th Year
Student 9	Female	20	3rd Year
Student 10	Female	22	4th Year
Student 11	Female	18	1st Year
Student 12	Male	22	4th Year

**Table 4:** Demographic data of survey participants (*n* = 99).

Occupational Therapy Student	Male	Female	Total
**Year of Study**			
First year	6	24	30
Second year	13	10	23
Third year	3	22	2
Fourth year	4	17	21
**Total**	**26**	**73**	**99**

**Table 5:** Integrated themes and survey findings.

Qualitative Method	Quantitative Method
Adaptive responses	Areas of occupation and daily activities during the COVID-19 pandemic (Monday through Friday, Saturday and Sunday)
Multidimensional challenges	
Economic impact	
Online learning and favourite hobbies	Visual activities
Impact on lifestyle	Auditory, smell/taste, and movement activities
Impact on social life	Meaningful activities

## Data Availability

Data are available from the corresponding author upon reasonable request.
